# Short-Term Reproducibility of Twenty-Four-Hour Intraocular Pressure Curves in Untreated Patients with Primary Open-Angle Glaucoma and Ocular Hypertension

**DOI:** 10.1371/journal.pone.0140206

**Published:** 2015-10-14

**Authors:** Shuo Xu, Qin Jiao, Yu Cheng, Jie Sun, Qiong Lu, Yisheng Zhong

**Affiliations:** 1 Department of Ophthalmology, Ruijin Hospital Affiliated Medical School, Shanghai Jiaotong University, Shanghai, China; 2 Department of Ophthalmology, Ruijin Hospital Luwan Branch, Shanghai, China; Harvard Medical School, UNITED STATES

## Abstract

**Purpose:**

To assess the short-term day-to-day reproducibility of 24-hour intraocular pressure (IOP) curves in various respects in untreated primary open-angle glaucoma (POAG) and ocular hypertension (OHT) patients.

**Methods:**

47 subjects with POAG and 34 subjects with OHT underwent IOP measurements every 2 hours in both eyes for consecutive 48 hours by a non-contact tonometer (NCT). IOP values at each time point were recorded. Mean IOP, peak IOP, time difference of peak IOP between two days and IOP fluctuation were also calculated. Intraclass correlation coefficients (ICCs) and Bland-Altman plots were used to evaluate reproducibility.

**Results:**

ICCs of the entire IOP values for a complete 24-hour curve were 0.577 and 0.561 in POAG and OHT patients, respectively. ICCs of IOP values at different time points ranged from 0.384 (10am) to 0.686 (4am) in POAG patients and from 0.347 (6am) to 0.760 (4am) in OHT patients. ICCs of mean IOP, peak IOP and IOP fluctuation were respectively 0.832, 0.704, 0.367 in POAG patients and 0.867, 0.816 0.633 in OHT patients. Only 37.23% and 35.29% of the peak IOP time points appeared within the time difference of 2 hours in POAG and OHT patients, respectively, while 53.19% and 48.53% appeared within 4 hours in POAG and OHT patients, respectively.

**Conclusion:**

A 24-hour IOP curve in a single day is not highly reproducible in short-term and has limited use for evaluating individual IOP condition. Mean IOP and peak IOP for a 24-hour IOP curve are useful parameters in clinical follow-up, while IOP value at a certain time point, IOP fluctuation and peak IOP time point should be interpreted with caution.

## Introduction

It is considered that elevated intraocular pressure (IOP) is the major risk factor for the development and progression of glaucoma. Decreasing IOP is recognized to be the most direct and efficient treatment strategy to prevent or retard the development of glaucoma. Mean IOP, peak IOP and IOP fluctuation have been respectively described as significant risk factors for progression of this disease [1–3]. In healthy population, the IOP fluctuation usually does not exceed 5 mmHg in one day, and have moderate circadian rhythms [4]. In patients with primary open-angle glaucoma (POAG) or ocular hypertension (OHT), IOP fluctuation is generally larger and circadian rhythms may be reversed [5]. On the contrary, several studies are controversial for the function of IOP fluctuation on the risk of the conversion from OHT to POAG or glaucomatous progression in the early glaucoma [6,7]. Nevertheless, these studies are variable because of different study objects and designs.

IOP values changes over time spontaneously. It has been presumed as tradition that there exists an inherent circadian IOP pattern and this circadian IOP pattern is reproducible and conserved from day to day in both glaucomatous and healthy population [8,9]. This assumption means that parameters of mean IOP, peak IOP and IOP fluctuation which have been speculated as important risk factors are relatively steady values from day to day with a given patient [10].

In clinical and research practices, 24-hour consecutive IOP measurements are used for glaucoma diagnosis, assessment of the IOP condition before or after a particular therapy, especially when a glaucoma patient is progressing. Of course, a complete 24-h IOP curve is not always feasible owing to convenience, time, and cost considerations. Even a simplified diurnal IOP curve with several measurements taken during office hours was alternatively used in clinical and research practices [11,12]. The measurements are generally limited in a single day based on the above presumption. In fact, recent studies do not fully support the presumption. Realini et al. [8,9] recently reported that both treated POAG patients and healthy individuals did not manifest a sustained and reproducible diurnal IOP pattern during office hours (from 8am to 8pm according to the studies) in the short term and drew the conclusion that single-day IOP measurements poorly characterize the diurnal IOP pattern. Song et al. [13] reached the conclusion that peak and valley IOP in 24-hour IOP curves showed excellent agreement while IOP fluctuation had poor reproducibility for a whole day in healthy young individuals. Aptel et al. [14] reported that patients with POAG did not manifest a reproducible diurnal IOP pattern during office hours (from 9am to 5pm according to the study) in the long term (from month to month). If 24-hour or diurnal IOP curves are not stable from day to day, the validity of the clinical and research practices based on this common presumption will be doubtable.

In contrast, Hatanaka et al. [15] reported the opposite conclusions which were conflicted with those of Realini et al. during office hours (from 8am to 4pm according to the study) in untreated POAG and OHT patients. And they also indicated that diurnal mean IOP, peak IOP and valley IOP had good circadian reproducibility and diurnal IOP fluctuation had only fair reproducibility in a single-day IOP curve [10]. It is no surprise that these studies provide the conflicting data on the reproducibility of 24-hour or diurnal IOP curves, perhaps because of the varied objects of studies, demographic characters, numbers and time of measurements, measuring instruments and antiglaucomatous treatment conditions. The purpose of our study is to assess the short-term day-to-day reproducibility of 24-hour IOP curves in POAG and OHT patients without antiglaucomatous treatment.

## Methods

### Patients

This was a prospective study which was conducted by the department of ophthalmology, Ruijin Hospital (Shanghai, China) and Ruijin Hospital Luwan branch (Shanghai, China) who recruited the hospitalized patients with ocular hypertension (OHT) and open-angle glaucoma (POAG) between December 2013 and April 2015. The study was approved by the Ethics Committee of Ruijin Hospital, Shanghai Jiaotong University (Shanghai, China), and performed in compliance with the principles of the Declaration of Helsinki. A written informed consent was necessary for any of the subjects before they were recruited in the study. Each subject underwent a consecutive 48-hour IOP curve measurement in both eyes during hospitalization.

The subjects included in the study were free of antiglaucomatous medicine treatment or had at least a 30-day medication washout period before measurement. Subjects with the history of any neurological or other ophthalmologic disease, any ophthalmology or intracranial surgeries, any ophthalmic laser treatment or any severe systemic disease were excluded from this study. Those who were less than 18 years old were also excluded. POAG was diagnosed according to the presence of typical glaucomatous abnormal optic nerve changes (localized optic disc notching, thinning or vertical cup-to-disc ratio asymmetry of > 0.2 between eyes, or localized wedge-shaped retinal nerve fiber layer defect) associated with matching glaucomatous visual field defects with standard automated perimetry (SAP), untreated IOP >21 mmHg with Goldmann applanation tonometer (GAT) in at least one eye, open angle on gonioscopy and no clinically apparent secondary cause for their glaucoma [16]. OHT was defined as having normal optic nerve head and retinal nerve fiber layer, normal visual field with SAP, untreated IOP >21 mmHg with GAT in at least one eye, open angle on gonioscopy and no clinically apparent secondary cause for high IOP [17].

### Measurements

Before the beginning of our study, the personal details were collected and recorded, including name, sex, age and ethnic. In order to detect ophthalmologic diseases, each subject underwent a series of ophthalmologic tests before 48-hour IOP curve measurement in both eyes, including best-corrected visual acuity, slit-lamp examination, Goldmann applanation tonometry, central corneal thickness (CCT), gonioscopy, ophthalmofundoscopy and visual field test. Diagnoses of subjects were based on the evaluation of experienced ophthalmologists. After undergoing these tests, the subjects finally had clinical classifications according to definitive diagnoses. Some of them were excluded due to discovery of other ophthalmologic abnormalities except for POAG and OHT.

White-on-white SAPs were conducted in visual field tests. We performed SAPs with two different kinds of automated perimeters because of the different instrument limitations in Ruijin Hospital and Ruijin Hospital Luwan Branch. Humphrey Visual Field Analyzer (HFA) II 750 (Carl Zeiss Meditec Inc., Germany) was used by means of Central 30–2 Threshold program with Swedish Interactive Threshold Algorithm Fast (SITA-Fast) strategy in Ruijin Hospital, while Octopus 101 automated perimeter (HAAG-STREIT Inc., Switzerland) was used in Ruijin Hospital Luwan branch by using G2 program with Dynamic strategy. Visual fields were judged to be abnormal if there existed a cluster of three or more adjacent test points with >5dB sensitivity reduction, or a cluster of two adjacent points with a lack of sensitivity >10dB (HFA in pattern deviation, Octopus automated perimeter in corrected probability) reduction compared to age-eccentricity corrected normal value [18]. If abnormal visual fields were confirmed, subjects were required to conduct computed tomography of the head to exclude neurological diseases.

CCT measurements were performed by an EM-3000 non-contact specular microscope (NCSM) (Tomey Inc., Japan). IOP measurements were taken every 2 hours for consecutive 48 hours in both eyes of the subjects during hospitalization. The time points of measurements were chosen as even numbers (e.g. 8am, 10am, 12am, 2pm, 4pm, 6pm, 8pm,10pm, 12pm, 2 am, 4am, 6am), and the first measurement was almost at 10am because it was usually the time when subjects began to hospitalize. IOP was measured in the sitting position with a calibrated AT550 auto non-contact tonometer (NCT) (Reichert Inc., USA) by experienced ophthalmologists and technicians. Especially, IOP was measured as quickly as possible after getting up during sleeping hours. Each measurement contained 3 times of IOP readings, and the average of 3 readings was recorded as the measurement result. Ophthalmologists and technicians were randomly exchanged between different days so that they were always masked to the measurement results of the previous day.

In order to reflect real daily-life IOP curves, patients were encouraged to keep their habitual daily schedules. Sleep cycles and activities of patients were not controlled. No food or drink was prohibited as well, including alcohol and caffeine.

### Statistical Analysis

The primary purpose of this study was to evaluate the short-term day-to-day reproducibility of 24-hour IOP curves in patients with POAG and OHT. Our data analyses included the following aspects of parameter: (1) reproducibility of IOP values at each time point between days (e.g., IOP at 2:00 AM in the first day compared with IOP at 2:00 AM in the second day); (2) reproducibility of the entire IOP values for a complete 24-hour IOP curve (12 measurement time points for each day) between days; (3) reproducibility of mean IOP between days; (4) reproducibility of peak IOP and peak IOP time points (defined as the maximum IOP value and the time point of appearance of peak IOP during one day, respectively) between days; (5) reproducibility of IOP fluctuation (defined as the maximum IOP value minus the minimum IOP value during the same day) between days.

In order to assess the reproducibility, the intra-class correlation coefficient (ICC) (with two-way random model and absolute agreement type) [19] and the Bland-Altman plot [20] were respectively used in our study. The following explanation for ICC has been described previously [21]: <0.4 represents poor agreement beyond chance; from 0.4 to 0.75 represents fair to good agreement beyond chance; and >0.75 represents excellent agreement beyond chance. Bland-Altman plots with mean differences and 95% limits of agreement (calculated as the mean difference of 2 methods ± 1.96 SD) were mainly used to evaluate the differences between individual measurements for each subject [22]. In this study, we made the hypothesis that there existed only one peak IOP time point in a certain day so as to obtain the unique value of peak IOP time point in one day. If the peak IOP values appeared at two or more than two time points in the same day, we compared the sum of IOP values of the two adjacent time points and chose the time point with the largest sum to be the peak IOP time point in one day [23]. Peak IOP time point of each day of each subject was compared with that of another day of the same subject. Peak IOP time points of a certain subject was considered as high reproducibility if the time difference of two peak IOP time points of this subject between two days was less than or equal to 2 hours. ICCs were calculated by using the software of Statistical Package for the Social Sciences version 21.0 (SPSS Inc.,Chicago, USA). Bland-Altman Plots were automatically established by using the software of Medcalc version 11.4.2.0 (Medcalc Software Inc., Mariakerke, Belgium).

## Result

**Fig 1:**
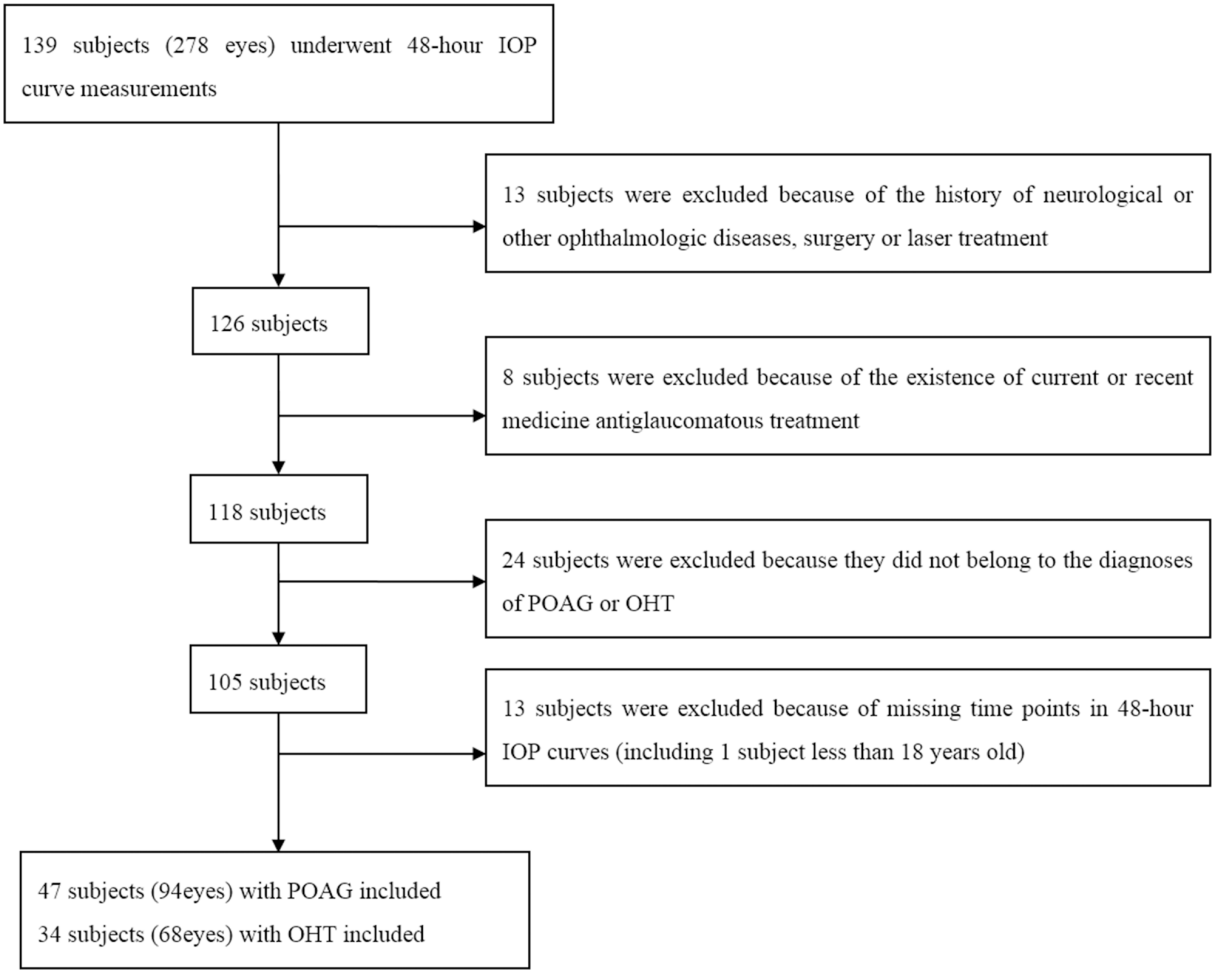
Flow Chart of the Selection Process.

A total of 81 subjects with 162 eyes were finally enrolled in this study. Among them, 47 were untreated POAG patients with 94 eyes and 34 were untreated OHT patients with 68 eyes. A flow diagram (Fig 1) showed the selection process of the subjects and the reasons for the exclusion. The mean age was 55.91±12.86 y (ranged from 21 to 87 y) in POAG group and 45.56±15.09 y (range from 21 to 70 y) in OHT group, respectively. More detailed demographic characteristics of the subjects were summarized in Table 1.

**Table 1:** Demographic Data of the Subjects.

Characteristic	POAG group	OHT group
Subject number	47	34
Eye number	94	68
Age (years) ^a^	55.91±12.86	45.56±15.09
Gender (male/female)	13/34	16/18
Race	100% East Asian	100% East Asian

^a^ Data are expressed as mean ± standard deviation (SD).

ICCs at each time point between two days were shown in Table 2. ICCs ranged from 0.384 (10am) to 0.686 (4am) in POAG group. Most of these values indicated fair to good agreement, with one value (0.384, 10am) indicating only poor agreement. In OHT group, ICCs ranged from 0.347 (6am) to 0.760 (4am), which represented the large variations of agreement from poor to excellent at different time points. However, the majority of the ICC values also demonstrated fair to good agreement. It was obvious that ICCs had a tendency to increase during bedtime (at 0am, 2am and 4am) in both groups.

**Table 2:** Mean IOP Values and ICCs at Each Time Point in/Between Two Days.

Time point	POAG group					OHT group				
	Day1 IOP value ^a^	Day2 IOP value ^a^	P value	ICC	95%CI	Day1 IOP value ^a^	Day2 IOP value ^a^	P value	ICC	95%CI
10am	20.86±2.79	20.08±3.10	0.020 ^b^	0.384	0.200–0.542	21.60±3.89	20.66±2.70	0.029 ^b^	0.452	0.244–0.621
12am	20.68±3.60	19.62±3.23	0.002 ^c^	0.553	0.383–0.685	20.86±3.15	19.87±3.18	0.023 ^b^	0.384	0.167–0.567
2pm	19.44±3.97	19.59±3.15	0.700	0.442	0.263–0.591	20.13±3.83	20.21±3.09	0.837	0.559	0.371–0.703
4pm	20.51±3.55	19.75±3.80	0.039 ^b^	0.535	0.375–0.665	19.92±3.38	20.16±3.30	0.55 7	0.469	0.261–0.636
6pm	19.39±3.59	19.24±3.26	0.658	0.547	0.388–0.674	20.32±3.73	20.33±3.10	0.969	0.597	0.419–0.731
8pm	18.66±3.11	18.40±3.13	0.388	0.564	0.410–0.688	19.30±3.48	19.38±4.12	0.880	0.405	0.184–0.586
10pm	18.81±3.47	18.38±3.96	0.252	0.526	0.363–0.657	20.39±4.44	19.56±3.86	0.106	0.484	0.282–0.646
0am	19.36±4.40	18.97±3.75	0.303	0.590	0.441–0.707	20.95±4.09	20.59±4.85	0.408	0.673	0.520–0.785
2am	20.07±4.01	19.63±4.57	0.227	0.676	0.550–0.772	21.27±3.76	21.00±4.14	0.481	0.681	0.529–0.790
4am	20.47±4.15	20.02±4.15	0.189	0.686	0.563–0.780	21.06±4.41	20.97±4.84	0.828	0.760	0.638–0.845
6am	19.92±3.77	20.00±3.39	0.806	0.587	0.437–0.705	21.61±3.97	21.37±3.89	0.668	0.347	0.118–0.540
8am	20.48±3.02	20.99±3.32	0.103	0.542	0.383–0.670	21.28±3.87	20.99±3.55	0.460	0.635	0.468–0.758

^a^ Data are expressed as mean ± SD;

^b^ P<0.05;

^c^ P<0.01.

**Fig 2:**
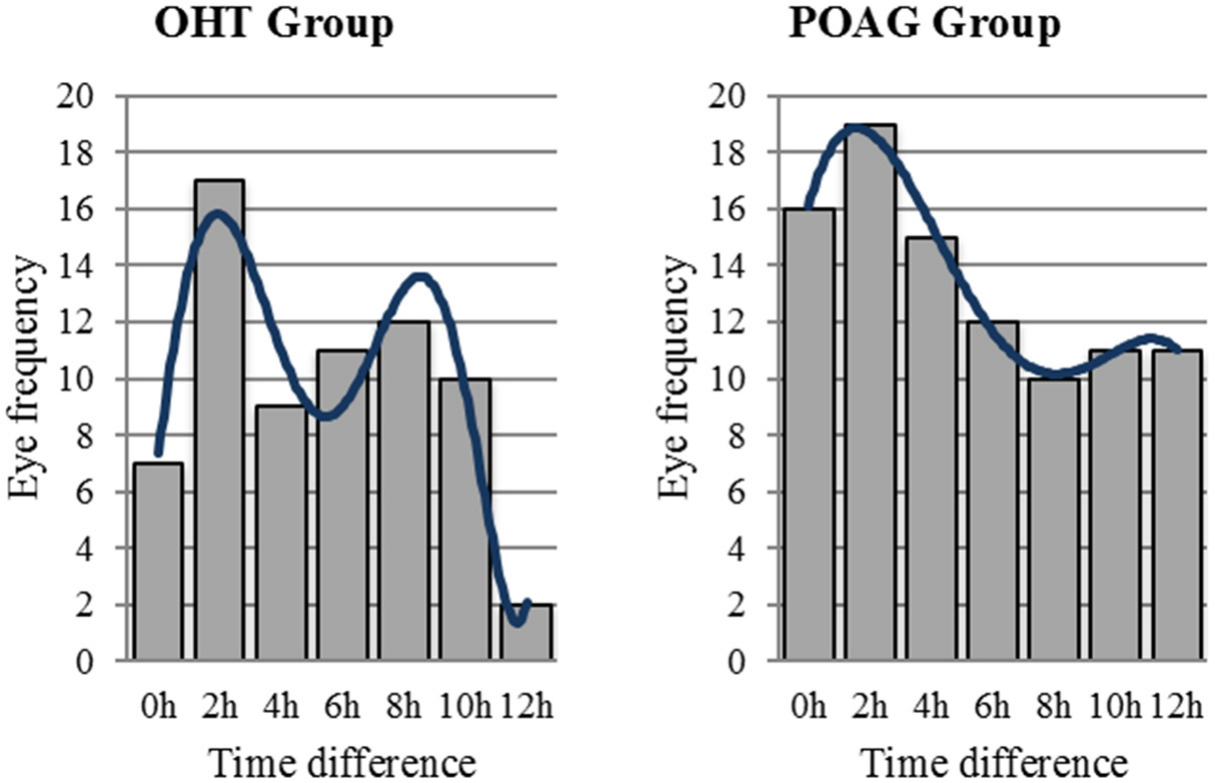
The Eye Frequency of the Time Difference of Peak IOP Time Points Between Two Days.

Table 3 showed the ICCs of main parameters between two 24-hour IOP curves. Mean IOP between two days showed excellent agreement with the ICC of 0.832 in POAG group and 0.867 in OHT group. Peak IOP had excellent reproducibility with the ICC of 0.816 in OHT group. The ICC of peak IOP in POAG group was 0.704, which represented only fair agreement. But it was closed to 0.75 and was obviously higher than those of IOP values for a complete 24-hour IOP curve (0.577 in POAG group and 0.561 in OHT group) and IOP fluctuation (0.367 in POAG group and 0.633 in OHT group) in both groups. However, peak IOP time points might greatly vary according to an unfixed pattern from day to day. Fig 2 was a frequency distribution plot counted with eye numbers. It showed the distribution of eye numbers in accordance with the time difference of peak IOP time points which was defined as time point of appearance of peak IOP during one day minus that of another day for a certain subject. According to our calculation, only 37.23% and 35.29% of the peak IOP time points appeared within the time difference of 2 hours in POAG and OHT group respectively, while only 53.19% and 48.53% appeared within 4 hours in POAG and OHT group, respectively.

**Table 3:** Parameters Values and ICCs of Diurnal IOP Curves in/Between Two Days.

Parameter	POAG group					OHT group				
	Day1 ^a^	Day2 ^a^	P value	ICC	95%CI	Day1 ^a^	Day2 ^a^	P value	ICC	95%CI
IOP value (for the whole 24h)	19.89±3.70	19.56±3.65	0.001 ^c^	0.577	0.536–0.615	20.72±3.89	20.42±3.80	0.018 ^b^	0.561	0.512–0.606
Mean IOP	19.89±2.71	19.56±2.65	0.039 ^b^	0.832	0.755–0.885	20.72±2.80	20.42±2.64	0.081	0.867	0.792–0.916
Peak IOP	24.38±3.60	24.09±3.20	0.295	0.704	0.586–0.793	25.62±3.90	25.60±4.01	0.960	0.816	0.718–0.882
IOP fluctuation	8.41±2.47	8.33±2.20	0.767	0.367	0.178–0.530	8.94±3.03	9.06±3.19	0.704	0.633	0.466–0.757

^a^ Data are expressed as mean ± SD;

^b^ P<0.05;

^c^ P<0.01.

IOP values for a complete 24-hour IOP curve had only fair reproducibility with the ICCs of 0.577 in POAG group and 0.561 in OHT group. ICC of IOP fluctuation in POAG group was 0.367, which indicated poor reproducibility. And it was remarkably lower than that in OHT group (0.633) which showed fair agreement.

**Fig 3:**
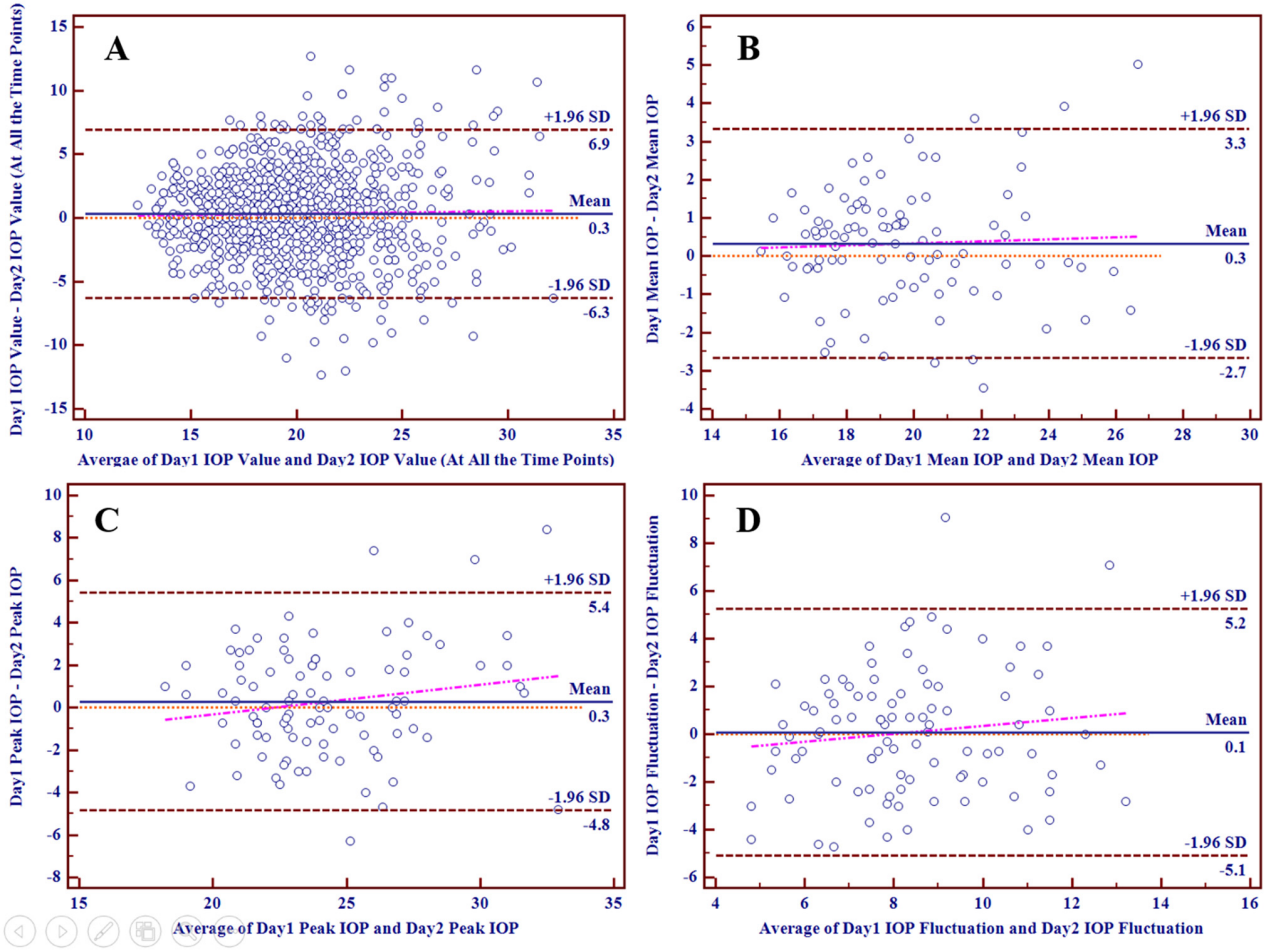
The Bland-Altman Plots for Different Parameters in POAG Group.

**Fig 4:**
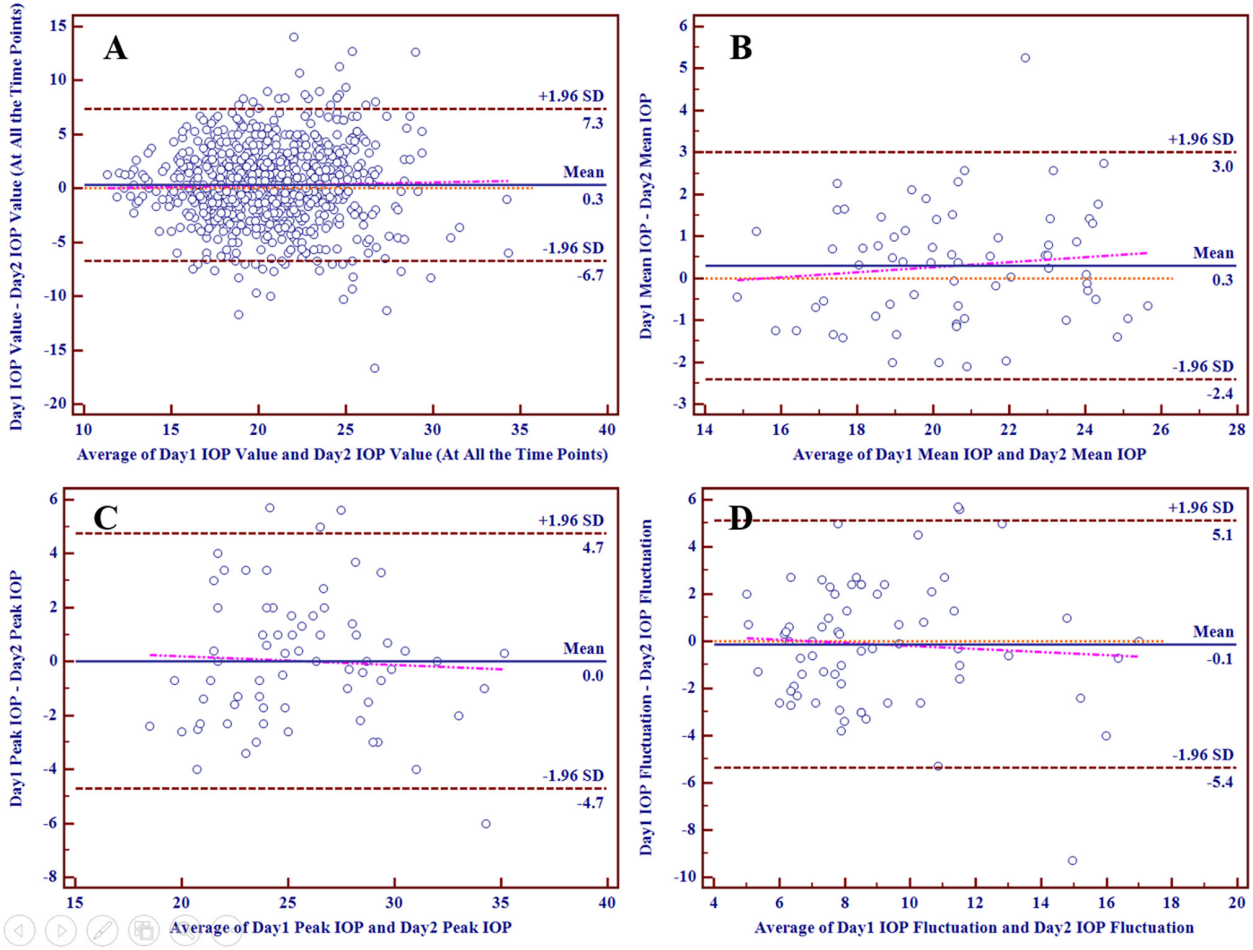
The Bland-Altman Plots for Different Parameters in OHT Group.

Figs 3 and 4 depicted the Bland-Altman plots comparing the 24-hour IOP curve parameters in individuals between two different days in POAG group and OHT group respectively, with mean differences and 95% limits of agreement provided. Part A, B, C and D respectively reflected the individual test-retest difference conditions of IOP values at all the time points in a 24-hour IOP curve, mean IOP, peak IOP and IOP fluctuation. The slopes of the scatter suggested that the differences of mean IOP, peak IOP and IOP fluctuation tended to increase slowly with increasing IOP values. In POAG group, the mean difference between two days was 0.3 mmHg for IOP values at all the time points in a 24-hour IOP curve, 0.3 mmHg for mean IOP, 0.3 mmHg for peak IOP and 0.1 mmHg for IOP fluctuation, while that in OHT group was 0.3 mmHg for IOP values, 0.3 mmHg for mean IOP, 0.0 mmHg for peak IOP and -0.1 mmHg for IOP fluctuation.

Individual test-retest differences of these parameters differed from one another. The proportions of test-retest differences of these parameters within different intervals were calculated and provided in Table 4. Intervals were established with various integers IOP values and 1.5 mmHg, because a difference of more than 1.5 mmHg in IOP was considered as clinically relevant [24]. Only mean IOP in both groups had high proportions of more than 50% (69.15% in POAG group and 76.47% in OHT group) within the difference of 1.5 mmHg. In both groups, more than 10% of IOP values at all the time points had test-retest differences of more than 5 mmHg, while almost 20% had those of more than 4 mmHg. In most cases, it was obvious that the proportions of mean IOP were significantly higher than those of the other parameters. On the contrary, the proportions of IOP values at all the time points in a 24-hour IOP curve were generally the lowest.

**Table 4:** Proportions of Parameter Test-Retest Differences Within Different Intervals Between Two Days.

Interval	POAG group				OHT group			
	IOP value (at all the time points) ^a^	Mean IOP ^a^	Peak IOP ^a^	IOP fluctuation ^a^	IOP value (at all the time points) ^a^	Mean IOP ^a^	Peak IOP ^a^	IOP fluctuation ^a^
≤1mmHg	29.26	53.19	35.11	34.04	29.78	52.94	38.24	35.29
≤1.5mmHg	38.21	69.15	44.68	41.49	37.87	76.47	47.06	44.12
≤2mmHg	51.51	80.85	59.57	56.38	50.61	85.29	61.76	52.94
≤3mmHg	69.33	93.62	77.66	79.79	65.56	98.53	80.88	83.82
≤4mmHg	80.05	98.94	92.55	89.36	77.57	98.53	94.12	89.71
≤5mmHg	87.15	98.94	95.74	97.87	85.66	98.53	95.59	94.12

^a^ Data are expressed as percentage.

## Discussion

In this study, we came to several main conclusions in 24-hour IOP curves that: (1) Mean IOP had excellent day-to-day reproducibility in both POAG and OHT patients; (2) Peak IOP was highly reproducible in OHT patients and had a tendency to be good reproducible in POAG patients, while the peak IOP time points had poor agreement in both POAG and OHT patients; (3) IOP fluctuation did not have high reproducibility in both POAG and OHT patients; (4) The entire IOP values for a complete 24-hour IOP curve had only fair to good reproducibility in both POAG and OHT patients; (5) IOP values at a certain time point had different reproducibility but tend to have only fair to good agreement in general in both POAG and OHT patients.

Part of the conclusions of our study were consistent with the finding of Hatanaka et al. [10], who reported that in OHT and POAG patients, the mean IOP and peak IOP had good reproducibility, whereas the reproducibility of IOP fluctuation was only fair. And another part of our conclusions were similar to those of Realini et al. [9], who reported that IOP did not manifest a repeatable diurnal pattern from day to day, except the difference that the subjects in their study were treated POAG patients. However, the part of our conclusions conflicted with another report of Hatanaka et al. [15], who reported that IOP followed a repeatable diurnal pattern in patients with untreated POAG and OHT. Opposite conclusion might be due to the different race compositions, different time point designs of measuring or different measuring instruments. It should be emphasized that in our study the race of subjects were totally East Asians because of the limitation of the local population, which made our study lack racial comparability to these recent studies. Diurnal IOP curves in waking hours or daytimes were general used to represent the whole 24-hour IOP curves in these studies. This might cause risk of misjudgement of the conclusions. The time point design of measurement which covered both daytime and bedtime was an advantage of our study. However, it might have influence on sleeping-awakening rhythms, even influence the IOP circadian rhythms. But a recent study reported that the 24-hour IOP rhythms seemed to be unaffected by hourly nocturnal awakening for IOP measurements in young healthy individuals [25]. Since IOP was generally higher in supine position [26–28] and because of the limitation of instruments (mobile tonometers had not been wildly used in local), IOP measurements were taken as quickly as possible after getting up to avoid a part of this factor. On the other hand, the main parameter to assess reproducibility of our study was ICC, which was defined as the ratio of the between-subject component of variance to the total variance. The same change tendencies in the same time points had only limited effect on reproducibility. In these recent studies, IOP was measured with GAT, while NCT was used in our study. Considering that the frequent IOP measurements (24 times in two consecutive days) with GAT might increase the potential risk of keratitis or corneal epithelial damage, we chose the NCT to conduct the study. It also reduced the requirements of the observers. Several studies reported that there was no significant difference between IOP obtained by GAT and NCT [24,29]. In a previous study, NCT was also used to evaluate coefficient of variation (COV) of IOPs in young Caucasians between two days by performing frequent IOP measurements (32 times in two successive daytimes) [30].

ICCs of the entire IOP values for a complete 24-hour curve and IOP fluctuation were lower than those of the other parameters. It might be due to that IOP values at the same time points between days were easily affected by many random factors, such as light exposure, activity or fluid intake, etc. As for IOP fluctuation, Hatanaka et al. [10] considered from a statistical perspective that IOP fluctuation involved more data points where there was inherently more variability than peak IOP, so the agreement would be generally poorer. Our study supposed that IOP fluctuation was far smaller values than peak IOP and mean IOP of one day (In our study, IOP fluctuation was generally about 1/3 of peak IOP and 1/2 of mean IOP), which expanded the influence of random errors in measurements. As for the high reproducibility of mean IOP, we supposed that mean IOP of one day neutralized part of the IOP variation over time, so mean IOP was more stable but represent less sensitively IOP changes over time than other parameters in our study.

**Fig 5:**
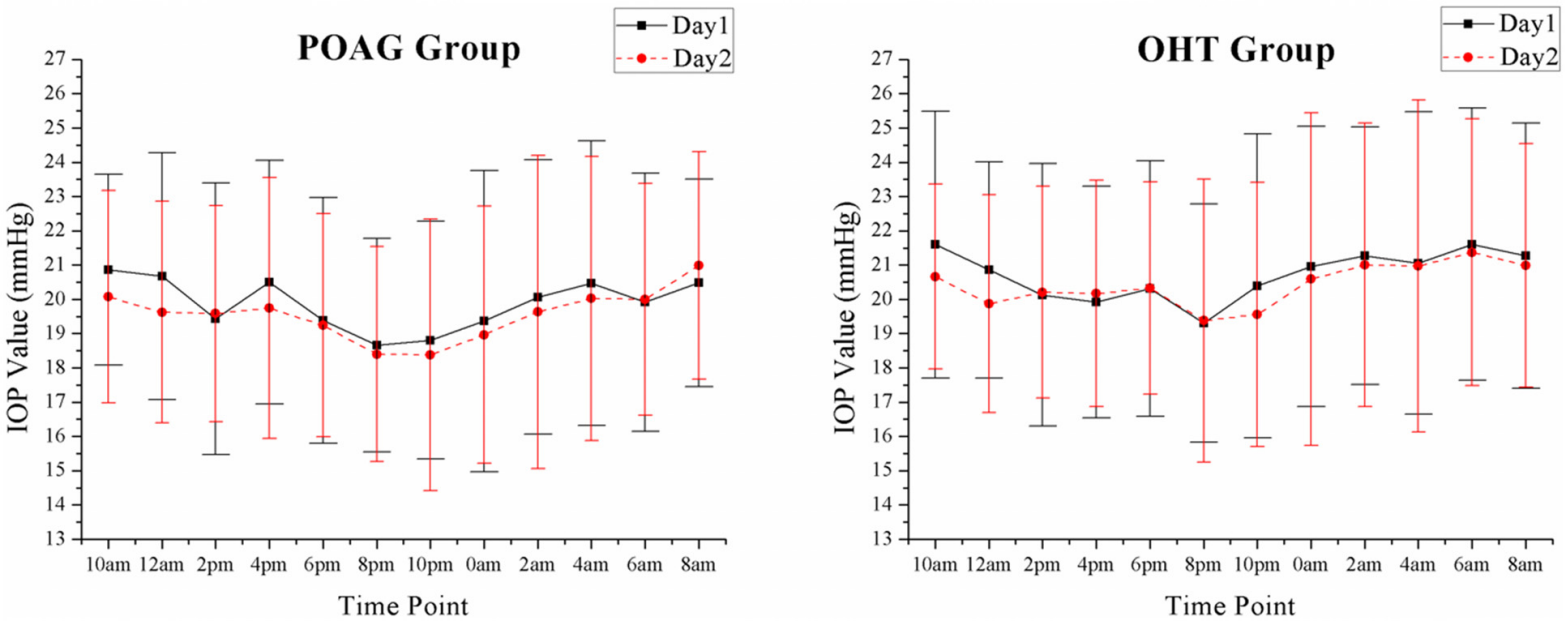
Twenty-Four-Hour IOP Curves in Two Days Described by Mean IOP Values at Different Time Points (Mean ± SD).

There were also some interesting phenomena in our study. ICCs had a tendency to increase during bedtime (at 0am, 2am and 4am) in both groups. A possible explanation was that the subjects' activities in the daytime following their own habits might increase the variability of IOP, while IOP might truly become relative stable values when they were asleep or in supine position or for a long time; Another possible explanation was that IOP was generally higher in supine position [26–28], which might make the influence of random errors in measurements relatively smaller and result in an increased ICCs. For another observation phenomenon, although Fig 5 showed the mean IOP values at different time points reached the peak values at the same time points (8am in POAG group and 6am in OHT group) in both two days, the analysis of individual time difference of peak IOP time points between two days showed that only almost half (53.19% and 48.53% in POAG and OHT group, respectively) of the peak IOP time points appeared within the time difference of 4 hours in both groups. This might be interpreted that POAG or OHT population followed a 24-hour or a closed-to-24-hour circadian rhythm, but there might exist great individual variation. Nevertheless, other possibilities could not be excluded by current studies. These phenomena need to be further researched.

It was worth noting that our study only conducted consecutive 48-hour IOP curve measurements. To obtain more data, longer consecutive IOP curve measurements are necessary. However, consecutive hospitalized IOP measurements are inconvenient, time-consuming and expensive for both subjects and researchers. In recent years, a wireless contact lens sensor (CLS) technique has been developed in order to monitor 24-hour IOP at home or in ambulatory conditions without the need for awakening subjects during sleep [31,32]. Some studies have shown its good safety and tolerability [33,34]. Others have shown its good reproducibility of measurements [35,36]. With this new technique, long-time consecutive IOP surveillances which reflect real IOP circadian rhythms will be more easily gained in clinical and research practices.

There were some limitations in our study. First of all, IOP was not corrected by CCT. However, Kida et al. [37] reported that there was no evidence that the 24-hour change in IOP was due to the changes in corneal biomechanical properties. Secondly, we did not control the factors of activities or amount and type of fluid and nutritional intake. Once again, some of the old subjects had concurrent chronic diseases which were not serious, such as diabetes and hypertension. And they were not prohibited from systemic medication uses. Finally, there existed certain factors we could not control and standardize between two days, such as temperature, humidity, light exposure and other possible environmental variables.

## Conclusions

In a word, this study demonstrates that a 24-hour IOP curve in a single day is not highly reproducible in short-term and has limited use for evaluating individual IOP condition. Mean IOP for a 24-hour IOP curve is highly reproducible. Peak IOP has less agreement than mean IOP, but still tend to be reproducible. On the contrary, IOP value at a certain time point and IOP fluctuation in general have relatively large variation and only fair reproducibility, or even poorer. So does the peak IOP time point. Mean IOP and peak IOP for a 24-hour IOP curve are useful parameters in clinical follow-up, while IOP value at a certain time point, IOP fluctuation and peak IOP time point should be interpreted with caution.

## Supporting Information

S1 Table The Individual Characteristic and IOP Information of Included Subjects. (XLSX)
